# Dynamic avoidance decision method for civil aircraft in a suborbital debris hazard zone

**DOI:** 10.1371/journal.pone.0289500

**Published:** 2023-08-01

**Authors:** Wantong Chen, Tianru Diao, Shiyu Ren, Shuguang Sun, Ruihua Liu

**Affiliations:** Department of Electronic Information and Automation, Civil Aviation University of China, Tianjin, China; Zonguldak Bülent Ecevit University: Zonguldak Bulent Ecevit Universitesi, TURKEY

## Abstract

Closing the static suborbital debris hazard zone method leads to low airspace resource utilization and long delays for civil aircraft, while the dynamic delineation of suborbital debris hazard zone method can solve the above phenomena. However, the existing research lacks the decision instruction for civil aircraft to avoid the dynamic suborbital debris hazard zone. To address the above problems, this paper creates probability ellipsoids of suborbital debris with different ballistic coefficients in the two-dimensional plane and use the divide-and-conquer algorithm for the dynamic delineation of the suborbital debris hazard zone. The suborbital debris hazard zone is extended outward by 10 km. Subsequently, the standard A* algorithm, the standard Lazy theta* algorithm, the improved Lazy theta* algorithm, and a flight path planning strategy are designed to avoid the suborbital debris hazard zone and provide safe dynamic avoidance commands for civil aircraft with fixed time intervals. The simulation results show that the average area of the dynamically delineated suborbital debris hazard zone is lower than the traditional static no-fly zone; the standard A* algorithm and improved Lazy theta* algorithm provides shorter flight path lengths and flight time and fewer waypoints in windless and windy conditions, respectively.

## 1 Introduction

In recent years, countries worldwide have vigorously developed their commercial space industries, resulting in an increased probability of launch failures. The global air traffic management system is facing unprecedented challenges due to the uncertainty surrounding accidents caused by suborbital vehicle disintegration during these launches. On the one hand, disintegration accidents in commercial space activities affect flight schedules in civil aviation airspace. During space launches, air traffic control usually restricts large areas of airspace in advance according to the launch plan. Affected civil aircraft reduce the risk of collision by changing their routes or delaying takeoff, but this also has consequences such as longer flight distances, increased delays, and fuel consumption [1, 2]. According to the American Airlines Pilots Association (ALPA), the launch of a Heavy Falcon rocket by Space Exploration Technologies at the Kennedy Space Center in Florida on February 6, 2018 affected airspace covering an area of 5,000 square nautical miles. This resulted in 563 flight disruptions, total delays of 4,645 minutes and an increase in total flight distance of 34,841 nautical miles. The incident had significant impact on airline interests [3]. Organizations such as China Aerospace Science and Technology Corporation (CASC) have also begun to develop and implement suborbital reusable vehicle test flight experiments. Additionally, the Airports Council International (ACI) predicts that civil aviation frequencies will increase to 22 million flights per year by 2025 [4]. Therefore, in dense commercial and civil aviation traffic networks, the use of traditional methods to delineate large airspace areas will result in strained airspace resources, increased control loads, and significant flight delays.

On the other hand, disintegration accidents in commercial spaceflight activities pose a serious risk to flight safety and crew. The high-risk space industry has long been dominated by the state, and private capital’s short-term technology accumulation is not yet sufficient to effectively control the risks of suborbital flights, resulting in a much higher accident rate for suborbital commercial flights than military space industry in various countries [5]. For example, in February 2003, the U.S. space shuttle Columbia unfortunately exploded and produced a large amount of debris as it was about to land; in December 2020, the SpaceX Starship full-size prototype SN8 underwent a suborbital test that culminated in an explosive disintegration; in September 2022, the suborbital New Shepard rocket launched by Blue Origin in West Texas urgently activated its abort system due to a failure shortly after liftoff. On April 20, 2023, SpaceX’s Starship launch vehicle failed to separate from its thrusters when its engines malfunctioned, causing it to explode mid-air. Suborbital vehicles fly at hypersonic speeds and are prone to disintegration, generating large amounts of debris under strong aerodynamic loads. In the atmospheric environment, the aerodynamic forces acting on the debris are characterized by randomness, making it difficult to accurately predict the process of debris falling. If such debris collides with a civil aircraft, it can cause instant catastrophic consequences [6]. Furthermore, in July 2022, China held the opening ceremony of the commercial launch site in Wenchang, Hainan, which will be expected to achieve regular launch in 2024. In view of the frequent suborbital launch activities in the future, how to create a safe and efficient decision support system for civilian aircraft to dynamically avoid suborbital danger zones has become a key research topic in civil aviation air traffic control.

At present, the emerging commercial space industry is at an early stage of development, and the research on suborbital debris area delineation and avoidance programs at home and abroad is still immature, while researchers in the United States, France and other countries have provided preliminary research guidance work in this field. For the problem of suborbital debris distribution, the United States, France and other countries have produced relevant software. Examples include the French launch and re-entry safety assessment tool ELECTRA© [7], the National Aeronautics and Space Administration’s (NASA) Common Real-Time Debris Footprint (CRTF) program [8], the Debris Risk Assessment (DeBRA) tool developed by APR Research [9], and Stanford University’s Range Safety Assessment Tool (RSAT) [10]. The source code of this software is limited to special user groups. The software programs are used to forecast the debris distribution and perform a ground risk assessment based on population databases. However, risk assessments of suborbital debris to ensure civil aviation safety are lacking. The Federal Aviation Administration (FAA) developed the Shuttle Hazard Area to Aircraft Calculator (SHAAC) [11] to assess the potential risk posed by the Space Shuttle to commercial aviation. However, the debris hazard area prediction module uses NASAs internal tool CRTF, and no other prediction methods have been published.

In the face of suborbital vehicle disintegration accidents, the National Airspace System (NAS) has typically used the traditional method of delineating a single rectangular shaped debris hazard zone. This method has led to a significant increase in the delay rate and flight time of civil aircraft [12]. Scholars in the United States have investigated suborbital debris hazard zone avoidance by civil aircraft. Researchers at Stanford University constructed a "streamlined envelope" of debris hazard zones based on the Future Air Traffic Management Concepts Evaluation Tool (FACET). This method reduces the interference and impact of suborbital launches on the national airspace system [13, 14]. NASA and FAA researchers have attempted to resolve potential conflicts between suborbital launches and civil airspace by constructing space transition corridors [15, 16]. The FAA pointed out the need to design decision support tools to assist controllers in rapid decision-making regarding flight avoidance in danger zones. FAA researchers, in conjunction with the Lincoln Laboratory, USA, developed a next-generation airborne collision avoidance system based on the Markov decision theory [17]. Researchers at Stanford University used this system and proposed an adaptive spatial discretization method for flight rerouting during space launches [18, 19]. Researchers at San Diego State University, USA, dynamically constructed a debris risk ranking graph based on risk tolerance and used the A* search algorithm based on a parallel computing framework to solve the path planning problem for flights, but their paths were not optimal [20, 21]. In terms of UAV avoidance of hazardous areas, Altan A’s team investigated neural network-based real-time control of a six-rotor UAV to minimize the error in the target load determined by path tracking [22]; used a population intelligence-based metaheuristic optimization algorithm to estimate the parameters of a quadrotor control algorithm to achieve path tracking as well as attitude and altitude control under different geometries such as rectangular, circular, and prismatic shapes [23]; and used a hybrid metaheuristic optimization algorithm of Harris Hawk optimization and Gray Wolf optimization to generate a fast and safe optimal path for the UAV [24]. The above approach investigates the path tracking and control of the vehicle, using strategies such as probabilistic risk analysis and parallel computational framework to delineate the dynamic flight restriction region. Although these methods are superior to static airspace restriction methods, they do not provide flight avoidance decision instructions to air traffic controllers and have limitations in practical applications. This study is intended to fill this gap.

Therefore, the purpose of this paper is to provide air traffic controllers with decision instructions for civilian aircraft to dynamically avoid suborbital debris hazard zones while reducing the path length, the number of waypoints, and the sum of the turning angles for civilian aircraft. We propose a dynamic decision method to enable a single civil aircraft to avoid a suborbital debris hazard zone at altitudes of 8–10 km. We create probability ellipsoids of the suborbital debris with different ballistic coefficients on a two-dimensional plane. The divide-and-conquer algorithm is used to dynamically delineate the suborbital debris hazard zone boundary and extend it to 10 km. To adapt to the dynamically changing suborbital debris hazard zone environment, a flight path planning strategy is designed to provide safe dynamic avoidance decision instructions at fixed time intervals, and the standard A* algorithm, the standard Lazy theta* algorithm, and the improved Lazy theta* algorithm are used to plan the path. The main contribution of this paper is to provide air traffic controllers with dynamic avoidance decision instructions for civil aircraft to avoid suborbital debris hazard zones, and the method is expected to be an important reference for exploring the collaboration model between commercial space and civil aviation in the future.

The remainder of this paper is organized as follows. Section 2 presents the problem description, assumptions, and the proposed model. Section 3 describes the standard A* algorithm, the standard Lazy theta* algorithm and the improved Lazy theta* algorithm for flight path planning. Section 4 presents the simulation results of the dynamically delineated suborbital debris hazard zone under windless and windy conditions and the comparison with the static no-fly zone delineated before the launch of the reusable suborbital vehicle. In addition, the standard A* algorithm, standard Lazy theta* algorithm, and improved Lazy theta* algorithm used for path planning are simulated and analyzed, and compared with the initial routes of civil aircraft and traditional methods of avoiding static no-fly zones, while providing a safe and efficient decision scheme for the dynamic avoidance of suborbital debris hazard zones for civil aircraft. Section 5 concludes the paper.

## 2 Problem modeling

### 2.1 Problem description and hypothesis

This paper focuses on a hypothetical scenario in which a suborbital reusable vehicle disintegrates at an altitude of 75 km above the Wenchang Satellite Launch Center in Hainan [25] and the debris falls into 3 million square kilometers of civil airspace [26]. The civil aircraft was cruising in the range of 8–10 km above sea level. In this paper, the northeast sky coordinate system is used to study the solution for civil aircraft to dynamically avoid the suborbital debris hazard zone. In order to ensure that civil aircraft safely avoid the dynamic suborbital debris hazard zone, real-time dynamic path planning is needed during the flight of civil aircraft to help the pilot of civil aircraft make the right decision, so the following assumptions are made in this paper:

- The motion state of the suborbital reusable vehicle from launch to the time of the disintegration accident can be observed by radar [27]. The initial position of the suborbital reusable vehicle at the moment of disintegration is (0,0,75), the time is 0 s, the initial heading angle is 0°, the initial track angle is -1°, the initial flight speed is 7.3 km/s, the initial longitude is 111°E, the initial latitude is 19°N, and the velocity increment of the debris in all directions at the moment of disintegration is 100 m/s.
- The air traffic controllers and civil aircraft pilots have real-time access to and can predict the status information of the reusable suborbital vehicle, the disintegration time of the debris, and the location of the debris hazard zone in the next 5 minutes so that they respond safely and rapidly.
- Only civil aircraft fly around the suborbital debris hazard zone and cannot cross the zone; therefore, we focus on dynamic path planning in a two-dimensional plane.
- The debris trajectory after the disintegration of the reusable suborbital vehicle is affected by the wind field, ballistic coefficient, and other factors. Therefore, we analyze the dynamic avoidance strategy of civil aircraft for different ballistic coefficients and with or without the wind field. The widely accepted Horizontal Wind Model 2014 (HWM14) is used to model the wind field [27].
- The debris hazard zone is represented by the probability ellipsoid of the suborbital debris projected on the two-dimensional plane, and it changes dynamically over time. Therefore, we dynamically limit the restricted airspace of the aircraft to reduce fuel consumption and improve airspace utilization.
- We conduct dynamic path planning of the civil aircraft in the cruise phase (an altitude of 8–10 km); therefore, it is assumed that the cruise speed remains constant at 800 km/h, and the pitch angle is zero.
- Debris smaller than 3 cm burns entirely as it falls. Therefore, we consider only debris larger than or equal to 3 cm.

### 2.2 Environmental modeling

#### 2.2.1 Suborbital debris hazard zone model

**Fig 1:**
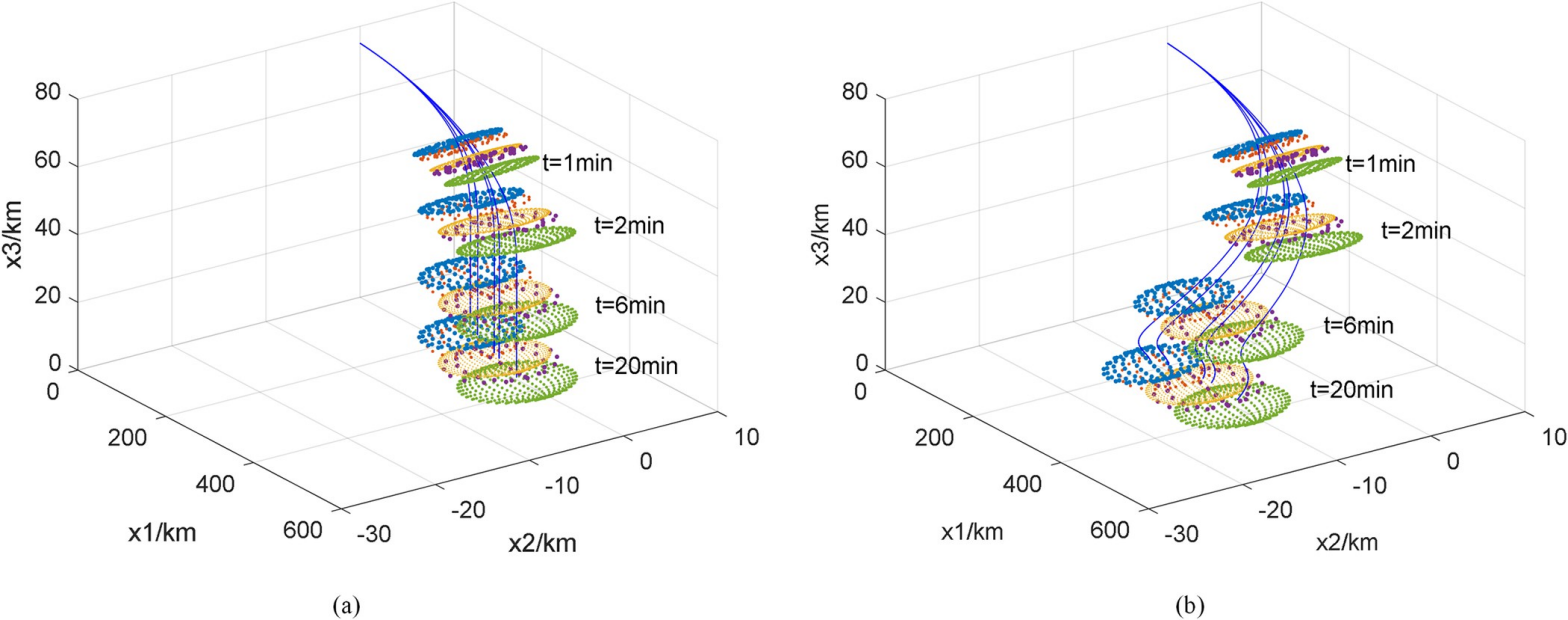
Debris trajectory in (a) windless conditions and (b) windy.

We used the covariance method to analyze the motion of individual fragments and created a prediction model of the debris trajectory [27], as shown in Fig 1. The debris was divided into five groups according to its size. The ballistic coefficients of the debris and other parameters are listed in Table 1. The size of the debris is denoted by *d*. After the reusable suborbital vehicle disintegrates, the debris with a ballistic coefficient of 11 kg/m^2^ first reaches 10 km altitude after 10.8 min. The last debris reaches 8 km altitude after 20.5 min and has a ballistic coefficient of 3.7 kg/m^2^. Since air traffic controllers can predict the time and location of the debris for the next 5 min [14], we focus on the debris zone and dynamic path planning 350–1250 s after the suborbital vehicle has disintegrated to ensure that the air traffic managers have enough time to communicate with the civil aircraft pilots to avoid the danger zone.

**Table 1:** Parameters of different-sized debris.

Suborbital debris	Debris size categories			
	1	2	3	4	5
Debris Size /m	0.03≤*d*<0.05	0.05≤*d*<0.08	0.08≤*d*<0.11	0.11≤*d*<0.20	*d*≥0.20
Ballistic coefficient /(kg/m^2^)	6.5	11.0	3.7	4.4	7.3
Time to reach 10km altitude /min	13.7	10.8	17.8	16.4	13.0
Time to reach 8km altitude /min	15.8	12.4	20.5	18.9	14.9
Time to reach the ground /min	26.4	20.5	35.4	31.9	25
Color	Yellow	Green	Blue	Red	Purple

#### 2.2.2 Strategies for delineating and expanding the boundaries of the restricted airspace

When hazardous weather, military activities, space launches, and other activities affect flight conditions, the air traffic control department temporarily designates areas of restricted airspace at various altitudes that cannot be entered by aircraft [28, 29]. The restricted airspace is typically defined as a two-dimensional convex or concave polygon [30]. However, there is a risk that aircraft may enter the suborbital debris hazard zone if they fly along the boundary of a concave polygon.

**Fig 2:**
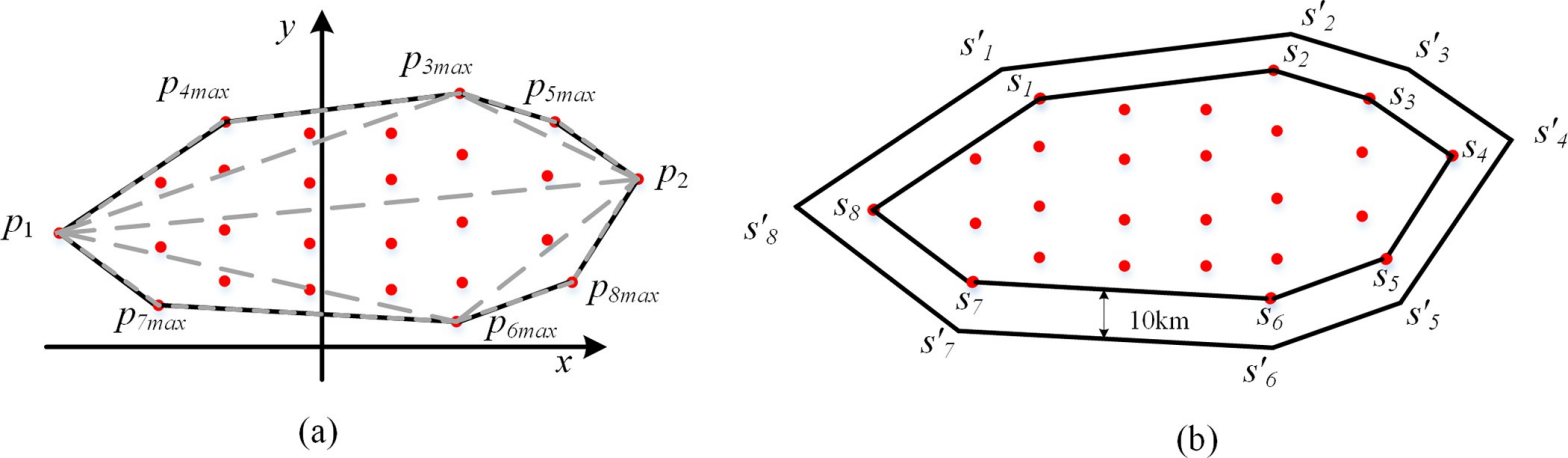
Schematic diagram of the delineation and extension of the boundary of the restricted airspace.

Therefore, we derive the probability ellipsoid of the debris hazard zone based on the acceptable risk for civil aviation [31]. We project the probability ellipsoid from the three-dimensional space to the two-dimensional plane and use the divide-and-conquer algorithm to delineate a convex polygon encompassing the projected points of the suborbital debris. This polygon represents the initial restricted airspace, reducing the risk of aircraft to encountering suborbital debris, as shown in Fig 2(A). The steps of the divide-and-conquer algorithm are as follows.

1. The debris with the smallest transverse coordinates is *p*_1_, and that with the largest transverse coordinates is *p*_2_.
2. Points *p*_1_ and *p*_2_ are connected. The area above *p*_1_*p*_2_ is referred to as the upper debris area, and that below the line is called the lower debris area. The set p of the vertices of the convex is expanded by the addition of the points *p*_1_ and *p*_2_.
3. In the upper debris area, the algorithm finds the point *p*_3max_, which has the farthest distance from *p*_1_*p*_2_. The point is added to the set *p*. Points *p*_1_, *p*_3max_, and points *p*_2_, *p*_3max_ are connected by lines. Areas above the lines *p*_1_*p*_3max_ and *p*_2_*p*_3max_ are defined as the upper debris areas (the same for the lower debris area). Step (3) is repeated until the number of debris pieces in the upper debris area is less than or equal to 1.
4. The same strategy as in Step (3) is used for the lower debris area.

At last, a safety buffer is required around the polygon surrounding the debris to minimize the risk of collision. Article 15 of Chapter 2 of the Airspace Management of the Basic Principles of Flight of the People’s Republic of China stipulates that the width of the flight path is 20 km, with 10 km on each side of the centerline. Therefore, we extend the initial airspace boundary by 10 km. Fig 2(B) shows the schematic diagram of the initial airspace boundary and the safety buffer. The boundary points of the initial restricted airspace are denoted as *S*_*i*_, and those of the extended restricted airspace are represented by $S_{i}^{'}$.

## 3 Path planning methods

### 3.1 The standard A* algorithm

The standard A* algorithm is a heuristic search algorithm which finds the global shortest path. The algorithm takes into account both the actual and predicted costs, and the standard evaluation function is as follows:

f(n)=g(n)+h(n) (1)

where *n* denotes a node; *f*(*n*) is the minimum cost function from the initial point (*x*_*start*_, *y*_*start*_) to the target point (*x*_*end*_, *y*_*end*_); *g*(*n*) is the cost function from the initial point (*x*_*start*_, *y*_*start*_) to the current point (*x*_*current*_, *y*_*current*_), which is calculated using the Euclidean distance; *h*(*n*) is the estimated cost function from the current point (*x*_*current*_, *y*_*current*_) to the target point (*x*_*end*_, *y*_*end*_). It is calculated using the Euclidean distance.

Since the A* algorithm search process uses a fixed-angle search mode, its search path is more tortuous and has more inflection points when the distribution of obstacles in the environment is complex.

### 3.2 The standard Lazy theta* algorithm

The standard Lazy theta* algorithm is a path planning algorithm using arbitrary angle search, which has the advantages of fewer inflection points, smoother paths and fewer search angles [32]. It overcomes the shortcomings of the standard A* algorithm with tortuous paths and many path inflection points. The standard Lazy theta* algorithm has the following process in Table 2. Its standard evaluation function is shown in Eq (1).

**Table 2:** The standard Lazy theta* algorithm.

Algorithm 1: The Standard Lazy Theta* Algorithm
**Step 1:** Input the suborbital debris hazard zone environment model; initialize the start and target points of the civil aircraft; create the OPEN and CLOSE tables, and add the start point to the OPEN table.
**Step 2:** Determine whether the OPEN table is empty. If it is empty, it means the pathfinding fails; otherwise, the node with the smallest evaluation function value in the OPEN table is taken as the current node to be expanded and set to *n*.
**Step 3:** Determine whether there is a line of sight between node *n* and the parent node of node *n*. If it exists, go to Step 4; otherwise, in the intersection of the CLOSE table with the neighboring nodes of node *n*, select the node with the lowest actual cost *f*(*n*) as the new parent node and update the cost of node *n*.
**Step 4:** Determine whether node *n* is the target point, and if so, the pathfinding is successful; otherwise, go to Step 5.
**Step 5:** Set all the neighbor nodes of the current node n to be extended to *m*.
**(1)** If node *m* is an obstacle or already exists in the CLOSE table, nothing is done.
**(2)** If node *m* is not in the OPEN table, add it to the OPEN table, set the parent node of node *m* to node *n*, and calculate the cost of node *m*.
**(3)** If node p is already in the OPEN table, determine if the current node *n* requires a lower cost than the original cost. If so, change the parent of node *m* to node n and update the cost of node *m*.
**Step 6:** Remove node *n* from the OPEN table and add it to the CLOSE table and return to Step 2.

Both the standard A* algorithm and the standard Lazy theta* algorithm have two deficiencies:

1. The evaluation function of the algorithm only considers the distances between the current position and the starting point and the target but does not take into account the distance between the current position and the suborbital debris hazard zone.
2. During the search process, the starting and target nodes of the algorithm are fixed; therefore, this algorithm is not applicable to dynamic environment path planning.

### 3.3 Improved Lazy theta* algorithm

#### 3.3.1 Risk-cost function

Based on Table 2, we added a risk cost function to the standard evaluation function in order to consider the distance between the current position and the suborbital debris hazard zone. The improved evaluation function *f*(*n*) is defined as follows:

$$\begin{array}{l} f(n)=g(n)+h(n)+r(n)\\ g(n)=\sqrt{{\left(x_{current}-x_{start}\right)}^{2}+{\left(y_{current}-y_{start}\right)}^{2}}\\ h(n)=\sqrt{{\left(x_{end}-x_{current}\right)}^{2}+{\left(y_{end}-y_{current}\right)}^{2}}\\ d(n)=\sqrt{{\left(x_{center}-x_{cur\_adj}\right)}^{2}+{\left(y_{center}-y_{cur\_adj}\right)}^{2}}\\ r(n)=\left\{ \begin{array}{l} 0,d(n)>R_{L}\\ \sqrt{{\left(d(n)\right)}^{2}+R_{L}{}^{2}},d(n)\leq R_{L} \end{array}\right. \end{array}$$ (2)

where *n* denotes a node; *f*(*n*) is the minimum cost function from the initial point (*x*_*start*_, *y*_*start*_) to the target point (*x*_*end*_, *y*_*end*_); *g*(*n*) is the cost function from the initial point (*x*_*start*_, *y*_*start*_) to the current point (*x*_*current*_, *y*_*current*_), which is calculated using the Euclidean distance; *h*(*n*) is the estimated cost function from the current point (*x*_*current*_, *y*_*current*_) to the target point (*x*_*end*_, *y*_*end*_). It is calculated using the Euclidean distance; *d*(*n*) is the Euclidean distance from the center of the ellipse containing the debris (*x*_*center*_, *y*_*center*_) to the neighbor nodes of the current point (*x*_*cur*_*adj*_, *y*_*cur*_*adj*_); *R*_*L*_ is the longest radius of the ellipse; *r*(*n*) is the risk-cost function of the distance from the current point to the suborbital debris hazard zone. If *d*(*n*)>*R*_*L*_, *r*(*n*) is zero, indicating that the current re-routing point is in the safe region; if *d*(*n*)≤*R*_*L*_, *r*(*n*) is $\sqrt{{\left(d(n)\right)}^{2}+R_{L}{}^{2}}$, indicating that the current rerouting point is not in the safe region, and its risk cost must be increased. The value of *r*(*n*) is determined based on the relationship between the magnitude of *d*(*n*) and *R*_*L*_ during the search of the improved Lazy theta* algorithm.

### 3.4 Flight path planning strategy

**Fig 3:**
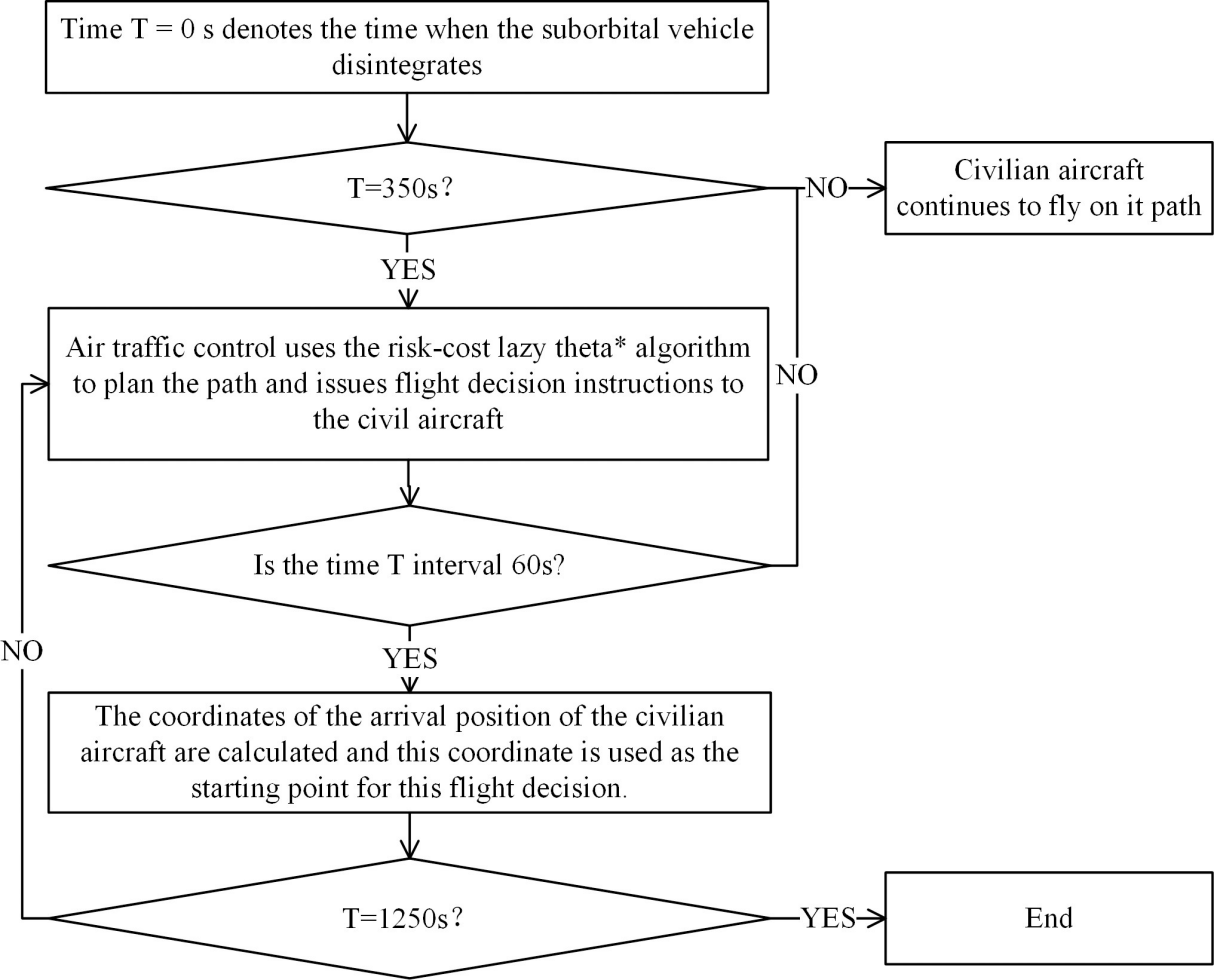
Flowchart of the path planning strategy.

To make the path planning algorithm better adapt to the dynamically changing environment, we propose a flight path planning strategy to avoid the dynamically delineated suborbital debris hazard zone by using variable starting points and fixed end points, as shown in Fig 3. The advantage of this strategy is that path planning with variable starting points and a fixed endpoint is performed every 60 s during the 350 to 1250 s period to ensure that air traffic controllers issue correct and safe instructions to civil aircraft every 60 s.

## 4 Simulation results and discussion

The simulation platform with an Intel® Core TM i7-10700 CPU @ 2.90GHz and 64.00 RAM using MATLAB R2019a to verify the performance and feasibility of the proposed method for the dynamic avoidance of suborbital debris in windless and windy conditions.

### 4.1 Dynamic delineation of suborbital debris hazard zone

**Fig 4:**
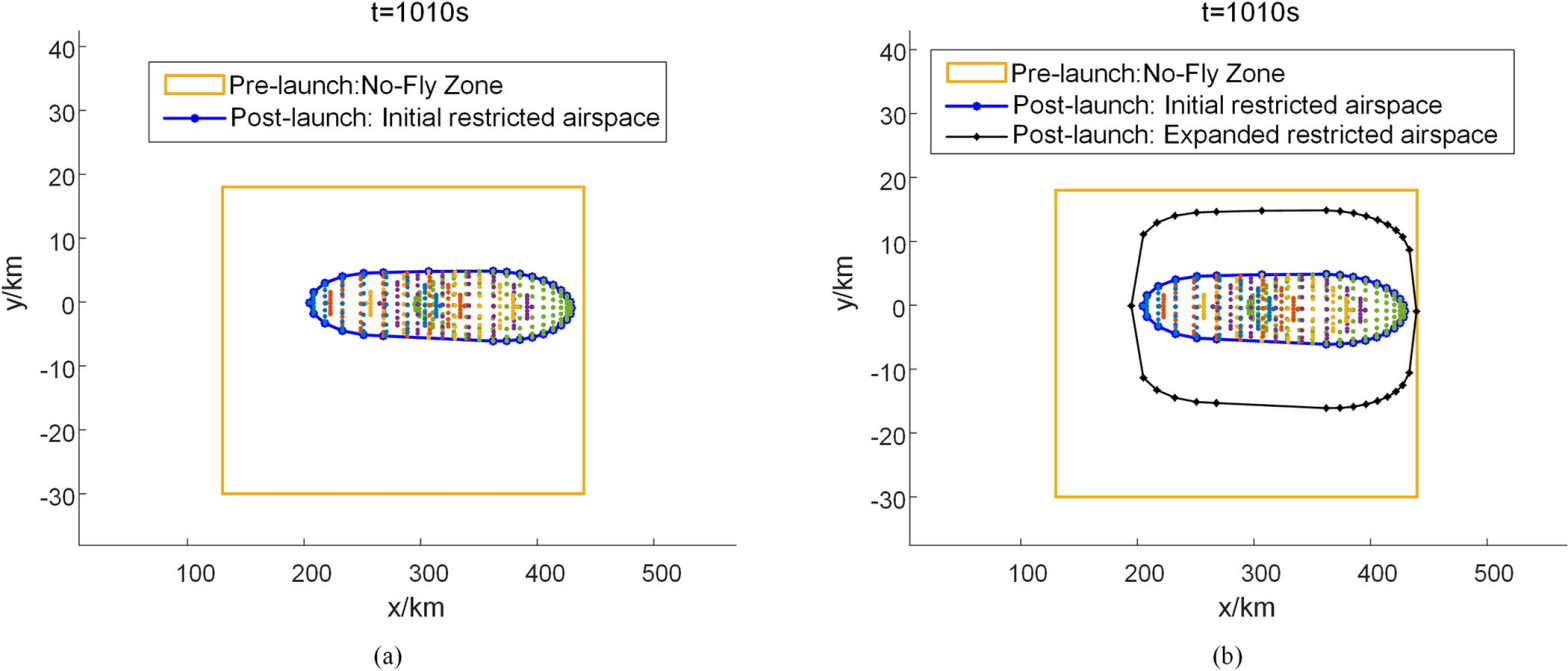
Schematic diagram of the suborbital debris hazard zone 1010 s after the vehicle’s disintegration under windless conditions.

**Fig 5:**
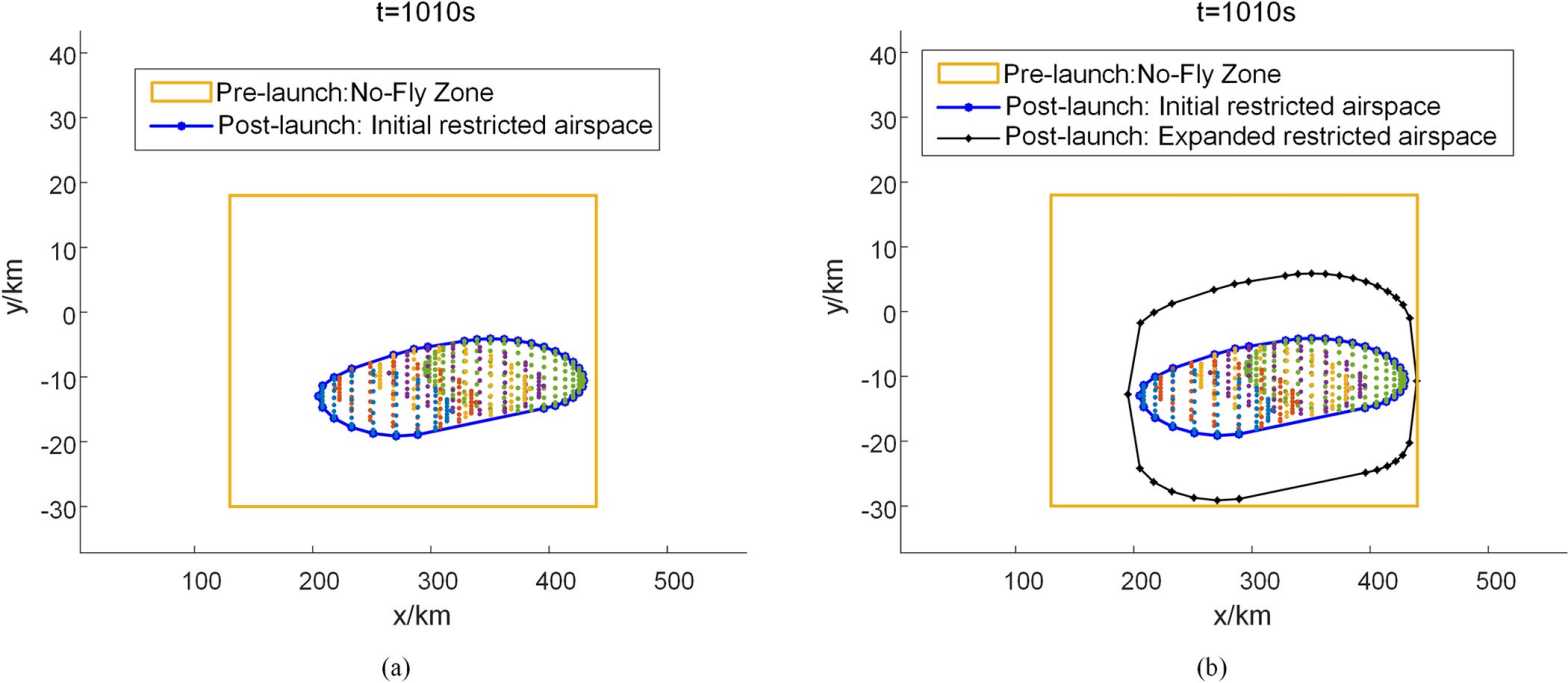
Schematic diagram of the suborbital debris hazard zone 1010 s after the vehicle’s disintegration under windy conditions.

**Fig 6:**
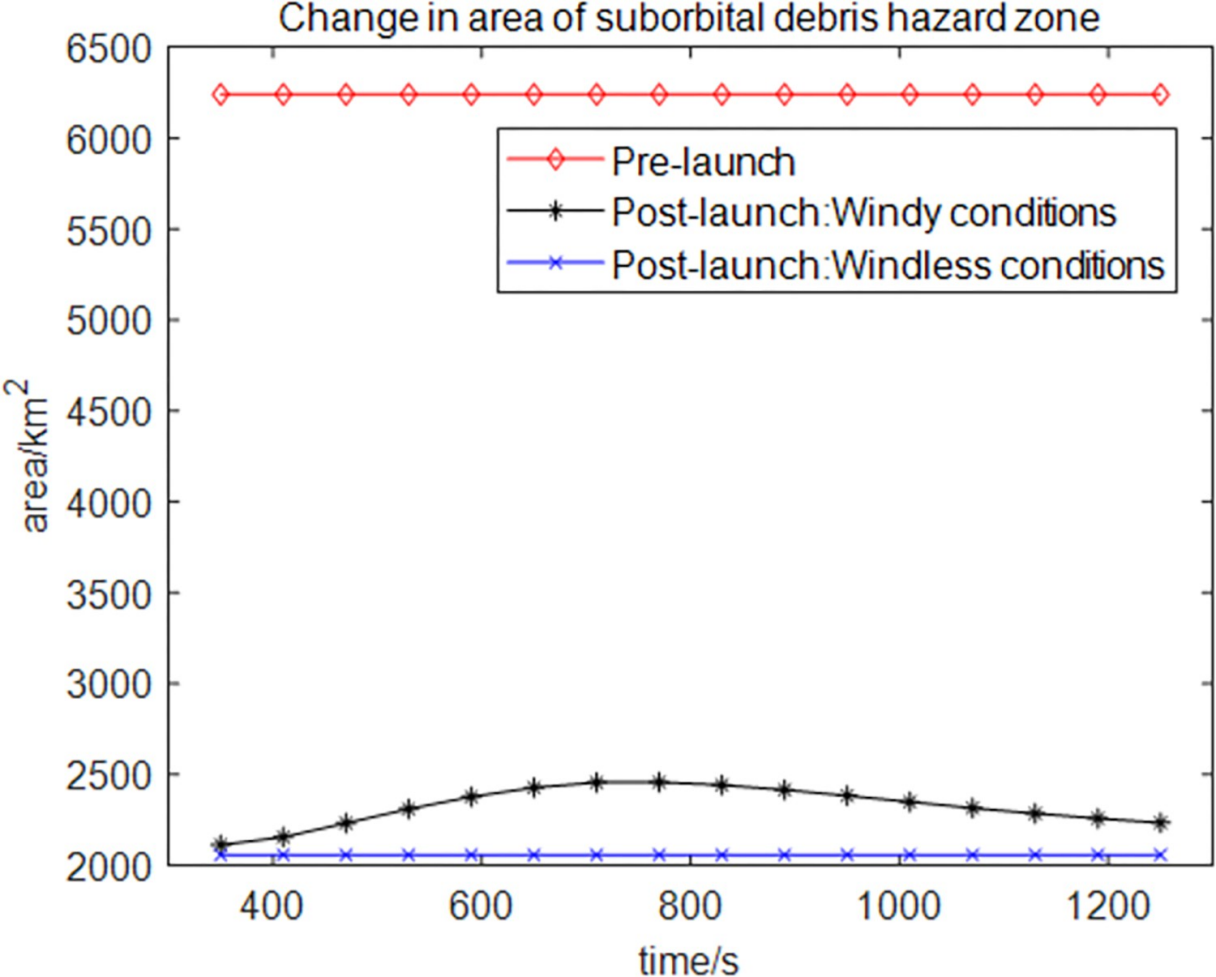
Change in the size of the restricted airspace.

It is assumed that the air traffic control department has designated a static no-fly zone (130 km×48 km) over the Wenchang Satellite Launch Center in Hainan before the launch of the reusable suborbital vehicle. Civil aircraft are prohibited from crossing this no-fly zone for several hours. After the disintegration of the reusable suborbital vehicle, we use the Divide-and-Conquer algorithm for the dynamic delineation of the suborbital debris hazard zone and extend it by 10 km based on the initial risk zone boundary. Figs 4 and 5, respectively show the initial and expanded risk zone boundaries of the suborbital debris under windless and windy conditions 1010 s after the disintegration of the vehicle. Fig 6 shows the change in the size of the restricted airspace during the disintegration period (350 s to 1250 s). The static no-fly zone delineated before the launch of the reusable suborbital vehicle has the largest area. In contrast, the area of the dynamically delineated debris hazard zone under windless and windy conditions changes dynamically. The average area of the dynamically delineated zone under windless and windy conditions is 67% and 62.7% smaller, respectively than that of the static no-fly zone. The proposed method for the dynamic delineation of the suborbital debris hazard zone results in a significant reduction of the restricted airspace, a more efficient use of the airspace, and less flight delays.

### 4.2 Dynamic avoidance of suborbital debris hazard zone

It is assumed that the civil aircraft flies on the planned route (-230,-15)→(10,-10)→(250,-5)→(490,-10)→(730,-15), and the altitude and flight speed remain unchanged during the cruise phase. The air traffic control department predicts the velocity and location of the debris in the next 5 min. It sends an alert message to the civil aircraft, asking it to change the flight path. At this time, the coordinates of the civil aircraft are (10,-10), and the coordinates of the target point are (490,-10). During the 350–1250 s period, the air traffic controller must issue instructions for evasive action to the civil aircraft every 60 s, including the coordinates of the current point, the new direction of the flight path, the angle, and the distance.

Table 3 compares the evaluation indexes of the initial route of civil aircraft and the traditional method of closing the large area static no-fly zone [12]. The evaluation indexes include the average area of the restricted airspace, length of path, flight time, number of waypoints and the sum of the angles of different flight paths. Prior to the launch of a suborbital reusable vehicle, air traffic control usually statically restricts a large area of airspace based on the launch plan and lasts for several hours. Since the planned routes of civil aircraft cross the suborbital debris hazard zone, they need to avoid the static no-fly zone to reduce the risk of collision with suborbital reusable vehicles during flight. However, this traditional approach does not allow for efficient use of airspace and requires civil aircraft to fly around large static no-fly zones, resulting in a significant increase in flight distance for civil aircraft. Compared with the initial route of civil aircraft, the path length and flight time of civil aircraft in the traditional method are 2.90% longer, the number of waypoints is 3 times higher than the initial route, and the sum of the angles of different flight paths is 28.7 times higher than the initial route, as shown in Table 3. The increase in the path length of civil aircraft and the increase in the turning angle of civil aircraft will prolong the arrival time of civil aircraft, increase the fuel consumption of civil aircraft, and lead to the economic interests of airlines.

**Table 3:** Comparison between the initial route of civil aircraft and the traditional method of closing the large static no-fly zone.

Path planning methods	the initial route of civil aircraft	the traditional method of closing the large static no-fly zone
Average area of the restricted airspace /km^2^	0	6240
Length of path /km	480.104	494.018
Flight time /min	36.008	37.051
Number of waypoints	1	3
The sum of the angles of different flight paths /°	2.387	68.574
Delays caused by the diversion of the civilian aircraft /min	0	1.043

In order to effectively reduce the aircraft delays and fuel consumption caused by closing the large static no-fly zone, this algorithm dynamically delineates the suborbital debris danger zone and, based on this, compares the initial routes of civil aircraft, the traditional method of closing the large static no-fly zone [12], the standard A* algorithm [20, 21], the standard Lazy theta* algorithm [32], and the improved Lazy theta* algorithm in terms of their advantages and disadvantages.

Table 4 shows the results of the paths planned by the standard A* algorithm, the standard Lazy theta* algorithm, and the improved Lazy theta* algorithm for the civil aircraft flying from (10,-10) to (490,-10) in windless conditions. As shown in Tables 3 and 4, the length of path, number of waypoints and the sum of the angles of different flight paths, and flight times of civil aircraft planned by the standard Lazy theta* algorithm and the improved Lazy theta* algorithm are the same. Compared with the traditional method of closing the large static no-fly zone, the path length and flight time of the standard A* algorithm are 2.84% less, the number of waypoints is one-third of the traditional method of closing the large static no-fly zone, and the sum of the angles of different flight paths is 0.017 times of the traditional method of closing the large static no-fly zone; the path length and flight time of the standard Lazy theta* algorithm and the improved Lazy theta* algorithm are 2.81% less, the number of waypoints is 2.667 times that of the traditional method of closing the large area static no-fly zone, and the sum of the angles of different flight paths is 0.411 times that of the traditional method of closing the large area static no-fly zone. Therefore, in windless conditions, the standard A* algorithm, the standard Lazy theta* algorithm and the improved Lazy theta* algorithm all outperform the traditional method of closing the large static no-fly zone. During the period of 350s-1250s of suborbital reusable vehicle disintegration, both the standard Lazy theta* algorithm and the improved Lazy theta* algorithm use the arbitrary angle search method to plan the path dynamically, while the standard A* algorithm uses the fixed angle search method to plan the path dynamically. Therefore, compared with the standard A* algorithm, the standard Lazy theta* algorithm and the improved Lazy theta* algorithm have 8 times more the number of waypoints and 23.609 times more the sum of the angles of different flight paths than the standard A* algorithm, and the path length and flight time are 0.027% longer. In summary, in windless conditions, the sum of angles and the number of waypoints of the standard A* algorithm are much smaller than those of the traditional method of closing a large static no-fly zone, the standard Lazy theta* algorithm and the improved Lazy theta* algorithm. The path length and flight time of the standard A* algorithm, the standard Lazy theta* algorithm and the improved Lazy theta* algorithm are basically the same, and they are all smaller than those of the traditional method of closing the large static no-fly zone. Therefore, in windless conditions, the standard A* algorithm outperforms the traditional method of closing large static no-fly zones, the standard Lazy theta* algorithm, and the improved Lazy theta* algorithm.

**Table 4:** Comparison of path planning algorithms in windless conditions.

Path planning methods	Standard A* algorithm	Standard Lazy theta* algorithm	Improved Lazy theta* algorithm
Average area of the restricted airspace /km^2^	2058.791	2058.791	2058.791
Length of path /km	480	480.131	480.131
Flight time /min	36	36.010	36.010
Number of waypoints	1	8	8
The sum of the angles of different flight paths /°	1.194	28.189	28.189
Delays caused by the diversion of the civilian aircraft /min	0	0.02	0.02

Table 5 shows the results of the paths planned by the standard A* algorithm, the standard Lazy theta* algorithm, and the improved Lazy theta* algorithm for civil aircraft flying from (10,-10) to (490,-10) in windy conditions. As shown in Tables 3 and 5, the standard Lazy theta* algorithm and the improved Lazy theta* algorithm are basically the same in terms of path length and civil aircraft flight time; the number of waypoints of the standard Lazy theta* algorithm is twice that of the improved Lazy theta* algorithm, and the sum of the angles of different flight paths of the improved Lazy theta* algorithm is 58.692% less than that of the standard Lazy theta* algorithm. Compared with the traditional method of closing the large static no-fly zone, the path length and flight time of the standard A* algorithm are 0.732% less, the number of waypoints is 3.667 times of the traditional method of closing the large static no-fly zone, and the sum of angles is 6.328 times of the traditional method of closing the large static no-fly zone; the path length and flight time of the standard Lazy theta* algorithm are 2.66% less, the number of waypoints is twice that of the traditional method of closing the large static no-fly zone, and the sum of angles is 0.508 times that of the traditional method of closing the large static no-fly zone; the path length and flight time of the improved Lazy theta* algorithm are 2.64% less, the number of waypoints is the same as that of the traditional method of closing the large area static no-fly zone, and the sum of angles is 0.210 times higher than the traditional method of closing the large static no-fly zone. Therefore, in windy conditions, the standard A* algorithm, the standard Lazy theta* algorithm, and the improved Lazy theta* algorithm all outperform the traditional method of closing large static no-fly zones. Compared with the standard A* algorithm, the path length and flight time of the standard Lazy theta* algorithm are 1.94% less, the sum of angle is 91.97% less, and the number of waypoints is 45.45% less; the path length and flight time of the improved Lazy theta* algorithm are 1.92% less, the sum of angle is 96.68% less, and the number of waypoints is 72.73% less. In summary, the path length and flight time of the standard Lazy theta* algorithm and the improved Lazy theta* algorithm are much smaller than those of the traditional method of closing the large static no-fly zone and the standard A* algorithm, and the sum of angles and the number of waypoints of the improved Lazy theta* algorithm are smaller than those of the standard A* algorithm. Therefore, in windy conditions, the improved Lazy theta* algorithm outperforms the traditional method of closing the large static no-fly zone, the standard A* algorithm and the standard Lazy theta* algorithm in windy conditions, and it effectively reduces the number of waypoints, the sum of angles, and the path length.

**Table 5:** Comparison of path planning algorithms in windy conditions.

Path planning methods	Standard A* algorithm	Standard Lazy theta* algorithm	Improved Lazy theta* algorithm
Average area of the restricted airspace /km^2^	2320.430	2320.430	2320.430
Length of path /km	490.400	480.892	480.995
Flight time /min	36.780	36.067	36.075
Number of waypoints	11	6	3
The sum of the angles of different flight paths /°	433.962	34.836	14.390
Delays caused by the diversion of the civilian aircraft /min	0.772	0.059	0.067

**Fig 7:**
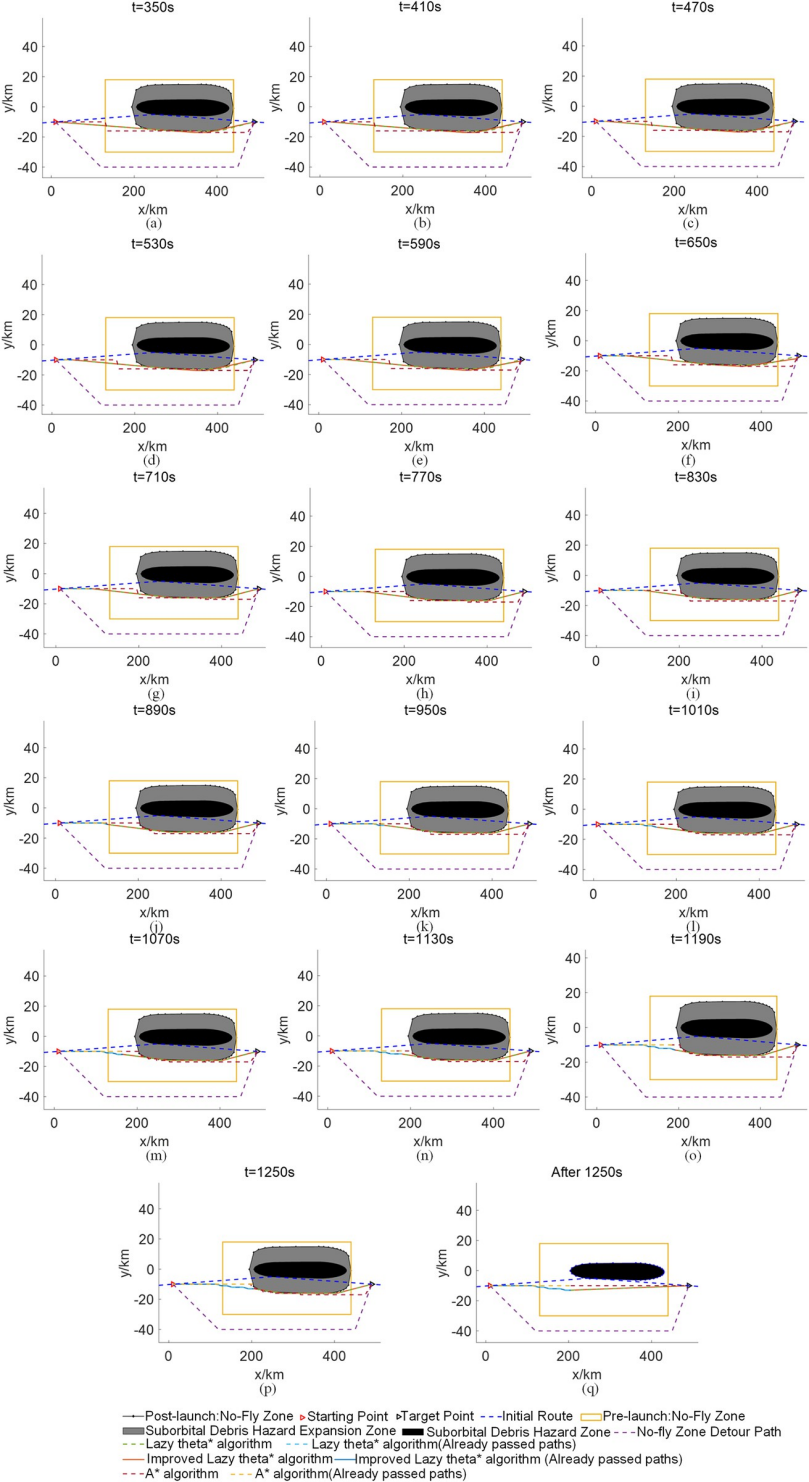
Path planning in windless conditions.

**Fig 8:**
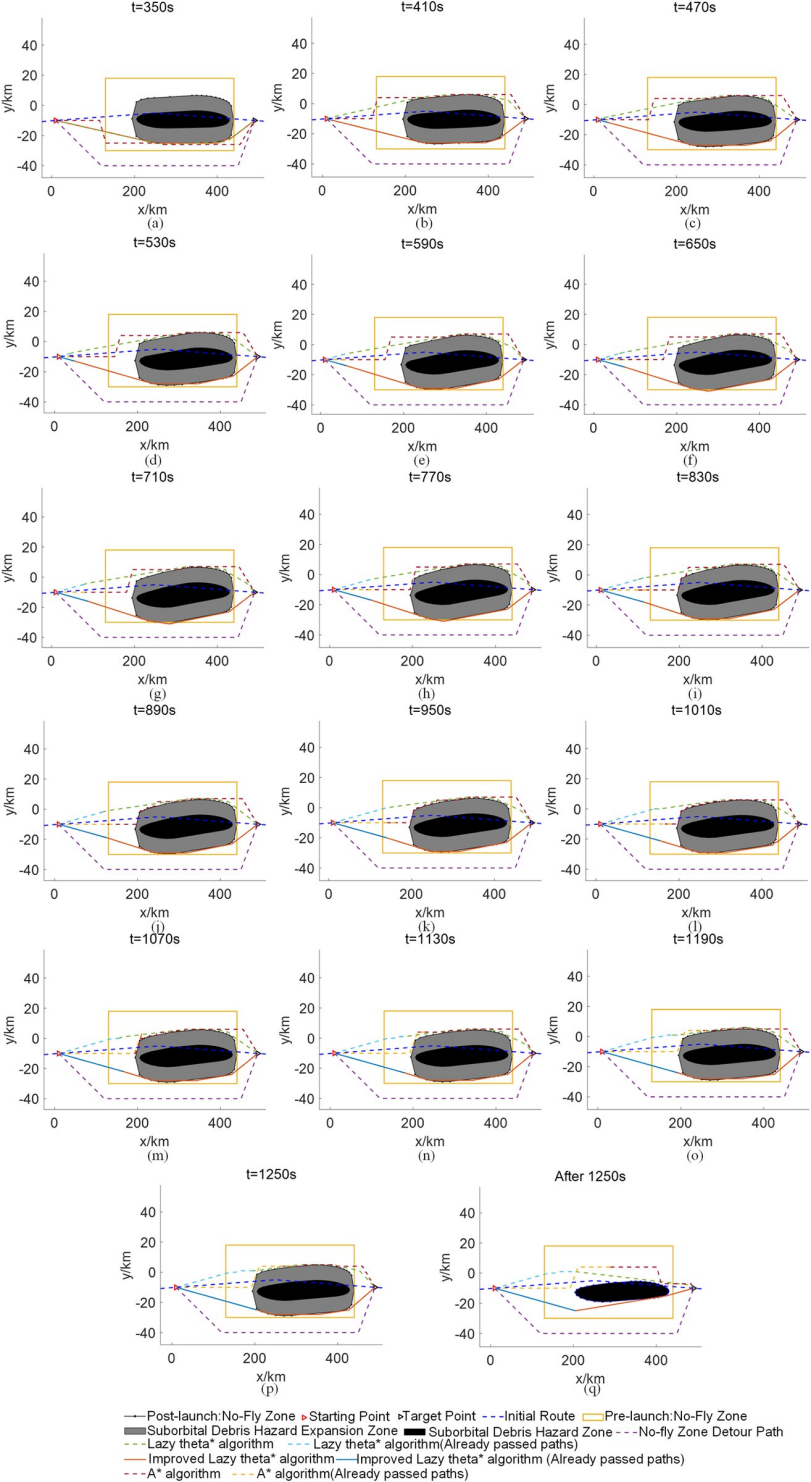
Path planning in windy conditions.

Fig 7 shows the civil aircraft path planning results for the initial route of civil aircraft in windless conditions, the traditional method of closing the large static no-fly zone, the standard A* algorithm, the standard Lazy theta* algorithm, and the improved Lazy theta* algorithm. Tables 6–8 show the decision schemes derived from the standard A* algorithm, the standard Lazy theta* algorithm, and the improved Lazy theta* algorithm for the dynamic avoidance of the suborbital debris hazard zone for civil aircraft in windless conditions, respectively. Fig 8 shows the civil aircraft path planning results for the initial civil aircraft route in windy conditions, the traditional method of closing the large static no-fly zone, the standard A* algorithm, the standard Lazy theta* algorithm, and the improved Lazy theta* algorithm. Tables 9–11 show the decision schemes for civil aircraft dynamic avoidance of the suborbital debris hazard zone derived using the standard A* algorithm, the standard Lazy theta* algorithm, and the improved Lazy theta* algorithm in windy conditions, respectively.

**Table 6:** Dynamic debris hazard zone avoidance decision scheme for A* algorithm in windless conditions.

Time	Area of restricted airspace /km^2^	A* algorithm			
		Location of waypoint	Direction of navigation	Angle of navigation /°	Flight distance /km
350s	2058.761	(10,-10)	To the right	1.194	13
410s	2058.77	(23,-10)	Unchanged	0	13
470s	2058.775	(36,-10)	Unchanged	0	13
530s	2058.78	(49,-10)	Unchanged	0	13
590s	2058.785	(62,-10)	Unchanged	0	13
650s	2058.788	(75,-10)	Unchanged	0	13
710s	2058.793	(88,-10)	Unchanged	0	13
770s	2058.791	(101,-10)	Unchanged	0	13
830s	2058.795	(114,-10)	Unchanged	0	13
890s	2058.796	(127,-10)	Unchanged	0	13
950s	2058.798	(140,-10)	Unchanged	0	13
1010s	2058.803	(153,-10)	Unchanged	0	13
1070s	2058.803	(166,-10)	Unchanged	0	13
1130s	2058.8	(179,-10)	Unchanged	0	13
1250s	2058.801	(192,-10)	Unchanged	0	13
1250s	2058.8	(205,-10)	Unchanged	0	285
After 1250s	decrease	No change in waypoint			

**Table 7:** Dynamic debris hazard zone avoidance decision scheme for standard Lazy theta * algorithm in windless conditions.

Time	Area of restricted airspace /km^2^	Standard Lazy theta* algorithm			
		Location of waypoint	Direction of navigation	Angle of navigation /°	Flight distance /km
350s	2058.761	(10,-10)	To the right	1.194	13
410s	2058.77	(23,-10)	Unchanged	0	13
470s	2058.775	(36,-10)	Unchanged	0	13
530s	2058.78	(49,-10)	Unchanged	0	13
590s	2058.785	(62,-10)	Unchanged	0	13
650s	2058.788	(75,-10)	Unchanged	0	13
710s	2058.793	(88,-10)	Unchanged	0	13
770s	2058.791	(101,-10)	Unchanged	0	13
830s	2058.795	(114,-10)	To the right	4.399	13.039
890s	2058.796	(127,-11)	To the left	4.399	13
950s	2058.798	(140,-11)	To the right	4.399	13.039
1010s	2058.803	(153,-12)	To the left	4.399	13
1070s	2058.803	(166,-12)	Unchanged	0	13
1130s	2058.8	(179,-12)	To the right	4.399	13.039
1250s	2058.801	(192,-13)	To the left	4.399	13
1250s	2058.8	(205,-13)	To the left	0.603	285.016
After 1250s	decrease	No change in waypoint			

**Table 8:** Dynamic debris hazard zone avoidance decision scheme for improved Lazy theta * algorithm in windless conditions.

Time	Area of restricted airspace /km^2^	Improved Lazy theta* algorithm			
		Location of waypoint	Direction of navigation	Angle of navigation /°	Flight distance /km
350s	2058.761	(10,-10)	To the right	1.194	13
410s	2058.77	(23,-10)	Unchanged	0	13
470s	2058.775	(36,-10)	Unchanged	0	13
530s	2058.78	(49,-10)	Unchanged	0	13
590s	2058.785	(62,-10)	Unchanged	0	13
650s	2058.788	(75,-10)	Unchanged	0	13
710s	2058.793	(88,-10)	Unchanged	0	13
770s	2058.791	(101,-10)	Unchanged	0	13
830s	2058.795	(114,-10)	To the right	4.399	13.039
890s	2058.796	(127,-11)	To the left	4.399	13
950s	2058.798	(140,-11)	To the right	4.399	13.039
1010s	2058.803	(153,-12)	To the left	4.399	13
1070s	2058.803	(166,-12)	Unchanged	0	13
1130s	2058.8	(179,-12)	To the right	4.399	13.039
1250s	2058.801	(192,-13)	To the left	4.399	13
1250s	2058.8	(205,-13)	To the left	0.603	285.016
After 1250s	decrease	No change in waypoint			

**Table 9:** Dynamic debris hazard zone avoidance decision scheme for A* algorithm in windy conditions.

Time	Area of restricted airspace /km^2^	A* algorithm			
		Location of waypoint	Direction of navigation	Angle of navigation /°	Flight distance /km
350s	2113.668	(10,-10)	To the right	1.194	13
410s	2156.512	(23,-10)	Unchanged	0	13
470s	2233.738	(36,-10)	Unchanged	0	13
530s	2311.188	(49,-10)	Unchanged	0	13
590s	2378.175	(62,-10)	Unchanged	0	13
650s	2429.211	(75,-10)	Unchanged	0	13
710s	2457.106	(88,-10)	Unchanged	0	13
770s	2458.614	(101,-10)	Unchanged	0	13
830s	2443.089	(127,-10)	Unchanged	0	26
890s	2416.625	(140,-10)	Unchanged	0	13
950s	2384.305	(166,-10)	Unchanged	0	26
1010s	2350.653	(192,-10)	To the left	36.384	19.925
1070s	2316.614	(211,4)	To the right	36.384	26
1130s	2286.233	(237,4)	Unchanged	0	26
1250s	2259.524	(263,4)	Unchanged	0	26
1250s	2235.709	(289,4)	Unchanged	0	12
After 1250s	decrease	(401,4)	To the right	45	11.402
		(412,-7)	To the left	45	72
		(484,-7)	To the right	45	1.414
		(485,-8)	To the left	45	1
		(486,-8)	To the right	45	1.414
		(487,-9)	To the left	45	1
		(488,-9)	To the right	45	1.414
		(489,-10)	To the left	45	1

**Table 10:** Dynamic debris hazard zone avoidance decision scheme for standard Lazy theta* algorithm in windy conditions.

Time	Area of restricted airspace /km^2^	Standard Lazy theta* algorithm			
		Location of waypoint	Direction of navigation	Angle of navigation /°	Flight distance /km
350s	2113.668	(10,-10)	To the left	3.205	13.038
410s	2156.512	(23,-9)	Unchanged	0	13.038
470s	2233.738	(36,-8)	Unchanged	0	13.038
530s	2311.188	(49,-7)	Unchanged	0	13.038
590s	2378.175	(62,-6)	Unchanged	0	13.038
650s	2429.211	(75,-5)	Unchanged	0	13.038
710s	2457.106	(88,-4)	Unchanged	0	13.038
770s	2458.614	(101,-3)	Unchanged	0	13.038
830s	2443.089	(114,-2)	Unchanged	0	13.038
890s	2416.625	(127,-1)	Unchanged	0	13.038
950s	2384.305	(140,0)	To the right	4.399	13
1010s	2350.653	(153,0)	To the left	4.399	13.038
1070s	2316.614	(166,1)	To the right	4.399	13
1130s	2286.233	(179,1)	Unchanged	0	13
1250s	2259.524	(192,1)	Unchanged	0	13
1250s	2235.709	(205,1)	To the right	1.848	279.145
After 1250s	decrease	(484,-8)	To the right	16.587	6.325

**Table 11:** Dynamic debris hazard zone avoidance decision scheme for improved Lazy theta* algorithm in windy conditions.

Time	Area of restricted airspace /km^2^	Improved Lazy theta* algorithm			
		Location of waypoint	Direction of navigation	Angle of navigation /°	Flight distance /km
350s	2113.668	(10,-10)	To the right	5.592	13.038
410s	2156.512	(23,-11)	Unchanged	0	13.038
470s	2233.738	(36,-12)	Unchanged	0	13.038
530s	2311.188	(49,-13)	Unchanged	0	13.038
590s	2378.175	(62,-14)	Unchanged	0	13.038
650s	2429.211	(75,-15)	Unchanged	0	13.038
710s	2457.106	(88,-16)	Unchanged	0	13.038
770s	2458.614	(101,-17)	Unchanged	0	13.038
830s	2443.089	(114,-18)	Unchanged	0	13.038
890s	2416.625	(127,-19)	Unchanged	0	13.038
950s	2384.305	(140,-20)	Unchanged	0	13.038
1010s	2350.653	(153,-21)	Unchanged	0	13.038
1070s	2316.614	(166,-22)	Unchanged	0	13.038
1130s	2286.233	(179,-23)	Unchanged	0	13.038
1250s	2259.524	(192,-24)	Unchanged	0	13.038
1250s	2235.709	(205,-25)	To the left	7.001	220.227
After 1250s	decrease	(425,-15)	To the left	1.796	65.192

In summary, although the traditional static no-fly zone method provides the safest solution for civil aircraft to avoid the suborbital debris hazard zone, it increases the fuel consumption of civil aircraft and the operating cost of airlines, therefore, this method is not the best decision method. The standard A* algorithm outperforms the other methods in terms of path length, flight time, the number of waypoints, and the sum of angles under windless conditions, but none of the indicators of the standard A* algorithm is optimal under windy conditions. Although the Lazy theta* algorithm has lower path lengths than the static no-fly zone method in windless and windy conditions, respectively, the number of waypoints and the sum of the angles are larger in windy conditions, and the safety risk is higher. In contrast, the improved Lazy theta* method can provide safe and efficient dynamic avoidance of suborbital debris hazard zone decision scheme for civil aircraft under windy conditions. Therefore, the use of standard A* algorithm and flight path planning strategy are the best method to dynamically avoid the suborbital debris hazard zone for civil aircraft under windless conditions, which can effectively reduce the problems of long path length, long flight time, large sum of redirected angles, and high fuel consumption caused by the large area of static no-fly airspace. Similarly, the use of improved Lazy theta* algorithm and flight path planning strategy are the best method to dynamically avoid the suborbital debris hazard zone for civil aircraft in windy conditions, which can also achieve this purpose.

## 5 Conclusion

This paper proposed a decision method for civil aircraft to avoid suborbital debris with different ballistic coefficients resulting from the disintegration of a suborbital vehicle. We discretized the suborbital debris trajectory and used a divide-and-conquer algorithm for the dynamic delineation of the suborbital debris hazard zone. We created five sets of probability ellipsoids of the suborbital debris in a two-dimensional plane and extended the hazard zone outward by 10 km. The restricted airspace was changed dynamically based on the location and velocity of the suborbital debris. We compared four decision methods for civil aircraft to avoid the suborbital debris hazard zone, including the static no-fly zone, the standard A* algorithm, the standard Lazy theta* algorithm, and the improved Lazy theta* algorithm. A flight path planning strategy was developed to provide instructions to the civil aircraft at a fixed interval to avoid the debris. The simulation results show that the standard A* algorithm and aircraft path planning strategy provide a more desirable decision solution in windless conditions, and the improved Lazy theta* algorithm and aircraft path planning strategy provide a more desirable decision solution in windy conditions. But we did not consider the flight dynamics of civil aircraft in this study. Therefore, more research is needed to optimize the routes for civil aircraft to avoid the suborbital debris hazard zone.
